# Supplementary material for: A COL7A1 Variant in a Litter of Neonatal Basset Hounds with Dystrophic Epidermolysis Bullosa
Source: Genes (Basel). 2020 Dec 4;11(12):1458. doi: 10.3390/genes11121458 (PMC7762066; doi:10.3390/genes11121458)
Supplement: Supplementary file 1 [file genes-11-01458-s001.zip › FigureS1_sequence.pdf]

Wildtype allele

```
     Primer F
1    gtgggagggc tatagggaag aacaattagg atatggggca ttgagggggc aggcaagagc
61   catgtcaaca cttctctctg acactagGTC CAGAGTCCAG CCAGACATTG CCCCCAGAAT
121  CCACGGCCAC AGATATCCTG GGGCTAAGGC CTGGAACCTC CTACCAGGTG GCTGTGTCAG
181  CACTGCGAGG GAGAGAGGAG AGTCCCCCTG TGGTCATCGT GGCTCAAACC Ggtcagggtc
241  tgacctagct ccttggctgg ctttctccag tggcccttta gacccctatg ccttccctct
301  cagactctca ctttgcccca caacagtctc cttcttcaga ttcccatgcc ttctccctca
361  gagtcctgct ttctcctttg gcctcccttt
                                   Primer R
```

Mutant allele

```
     Primer F
1    gtgggagggc tatagggaag aacaattagg atatggggca ttgagggggc aggcaagagc
61   catgtcaaca cttctctctg acactagGTC CAGAGTCCAG CCAGACATTG CCCCCAGAAT
121  CCACGGCCAC AGATATCCTG GGGCTAAGGC CTGGAACCTC CTACCAGGTG GCTGTGTCAG
181  CACTGCGAGG GAGAGAGGAG AGTCCCCCAT CGTGGCTCAA ACCGgtcagg gtctgaccta
241  gctccttggc tggctttctc cagtggccct ttagacccct GTGTCAGCAC TGCGAGGGAG
301  AGAGGAGAGT CCCCCATCGT GGCTCAAACC Ggtcagggtc tgacctagct ccttggctgg
361  ctttctccag tggcccttta gacccctatg ccttccctct cagactctca ctttgcccca
421  caacagtctc cttcttcaga ttcccatgcc ttctccctca gagtcctgct ttctcctttg
481  gcctcccttt
        Primer R
```

Figure S1. Genomic sequences of the wildtype and mutant alleles. The sequences correspond to the PCR amplicons used for genotyping the *COL7A1*:[c.2028_2034del; c.1993_2050+56dup] variant. The primer sequences are indicated in green. Intronic bases are given in lowercase letters, exonic bases are given in uppercase letters. The duplicated region is highlighted in grey and the 7 bp deletion within the duplicated region is underlined.
