# Supplementary material for: A COL7A1 Variant in a Litter of Neonatal Basset Hounds with Dystrophic Epidermolysis Bullosa
Source: Genes (Basel). 2020 Dec 4;11(12):1458. doi: 10.3390/genes11121458 (PMC7762066; doi:10.3390/genes11121458)
Supplement: Supplementary file 1 [file genes-11-01458-s001.zip › FigureS2_EM_Images.pdf]

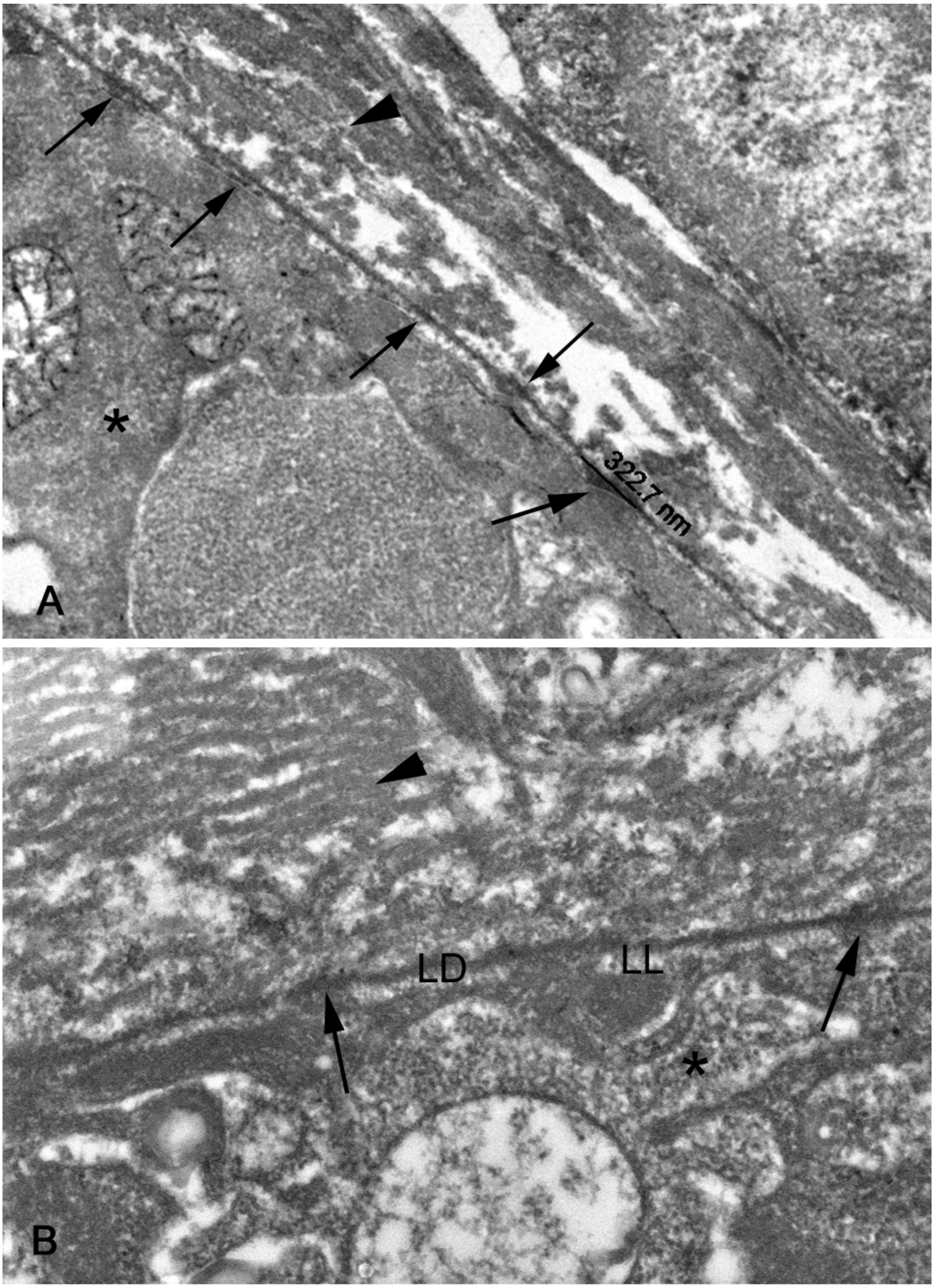

Figure S2. Ultrastructural exam of both the affected puppy (A) and the age-matched control (B) identified the intermediate filaments of the basal keratinocyte (*), collagenous matrix of superficial dermis (arrowheads) and hemidesmosomes (arrows). Regions of the lamina lucida (LL) and lamina densa (LD) were identified but visualization was impaired by autolysis. Hemidesmosomes in both puppies ranged from 269-447 nm in length and were distributed approximately every 716 nm along the basement membrane zone. Anchoring fibrils could not be visualized in either puppy.
